# Integration of genotypic, hyperspectral, and phenotypic data to improve biomass yield prediction in hybrid rye

**DOI:** 10.1007/s00122-020-03651-8

**Published:** 2020-07-17

**Authors:** Rodrigo José Galán, Angela-Maria Bernal-Vasquez, Christian Jebsen, Hans-Peter Piepho, Patrick Thorwarth, Philipp Steffan, Andres Gordillo, Thomas Miedaner

**Affiliations:** 1 State Plant Breeding Institute, University of Hohenheim, 70593 Stuttgart, Germany; 2 KWS SAAT SE, Grimsehlstraße 31, 37574 Einbeck, Germany; 3 Biostatistics Unit, Institute of Crop Science, University of Hohenheim, 70593 Stuttgart, Germany; 4 KWS LOCHOW GMBH, Ferdinand-von-Lochow Straße 5, 29303 Bergen, Germany

## Abstract

**Key message:**

Hyperspectral and genomic data are effective predictors of biomass yield in winter rye. Variable selection procedures can improve the informativeness of reflectance data.

**Abstract:**

Integrating cutting-edge technologies is imperative to sustainably breed crops for a growing global population. To predict dry matter yield (DMY) in winter rye (*Secale cereale* L.), we tested single-kernel models based on genomic (GBLUP) and hyperspectral reflectance-derived (HBLUP) relationship matrices, a multi-kernel model combining both matrices and a bivariate model fitted with plant height as a secondary trait. In total, 274 elite rye lines were genotyped using a 10 k-SNP array and phenotyped as testcrosses for DMY and plant height at four locations in Germany in two years (eight environments). Spectral data consisted of 400 discrete narrow bands ranging between 410 and 993 nm collected by an unmanned aerial vehicle (UAV) on two dates on each environment. To reduce data dimensionality, variable selection of bands was performed, resulting in the least absolute shrinkage and selection operator (Lasso) as the best method in terms of predictive abilities. The mean heritability of reflectance data was moderate ($h^{2}$ = 0.72) and highly variable across the spectrum. Correlations between DMY and single bands were generally significant (*p* < 0.05) but low (≤ 0.29). Across environments and training set (TRN) sizes, the bivariate model showed the highest prediction abilities (0.56–0.75), followed by the multi-kernel (0.45–0.71) and single-kernel (0.33–0.61) models. With reduced TRN, HBLUP performed better than GBLUP. The HBLUP model fitted with a set of selected bands was preferred. Within and across environments, prediction abilities increased with larger TRN. Our results suggest that in the era of digital breeding, the integration of high-throughput phenotyping and genomic selection is a promising strategy to achieve superior selection gains in hybrid rye.

**Electronic supplementary material:**

The online version of this article (10.1007/s00122-020-03651-8) contains supplementary material, which is available to authorized users.

## Introduction

The European biogas sector has attracted increasing attention as a renewable source of heat, electricity, and transport suitable for climate change mitigation with additional socioeconomic advantages (Scarlat et al. 2018). Political directives (European Renewable Energy Directive 2009/28/EC) supporting the production of bioenergy have already been implemented in Europe (European Union 2009). This legislation stated that, by 2020, the energy demanded in the European Union (EU) should be supplied in at least 20% by renewable sources. Among the EU members, therefore, the role of energy crops as bioenergy feedstocks has undergone a considerable increase, represented mainly by silage maize (European Commission 2018). Maize monocropping is, however, discouraged by regulations toward enhanced sustainability of the biomass production (European Union 2010).

Additionally, in Germany, the principal biogas producer in Europe, a limit was placed on the amount of maize acceptable in the fermentation substrate. In 2012, this limit was set to 60%, while in 2021, it will be reduced further to 44% (Renewable Energy Sources Act “EEG”; EEG 2012, 2017). Consequently, the growing demand for bioenergy combined with the search for alternative sources of biomass opens a very attractive opportunity for diversifying crop rotations.

Winter rye (*Secale cereale* L.) is a small-grain cereal with vigorous growth and enhanced tolerance to abiotic (e.g., low temperatures, light or acid soils with low fertility) and biotic stress factors. It can, therefore, be cultivated in vast areas less suited for other cereal crops (Geiger and Miedaner 2009), representing a sustainable biomass source with reduced competition with food or feed (Miedaner et al. 2012; Geiger and Miedaner 2009). Although it is present worldwide, rye is mostly grown in Northeastern Europe, where Germany, Poland, Russia, and Fennoscandia concentrate about 60% of the total area of rye cultivation (FAO 2019). Considering its potential as a dual-purpose crop, enhanced dry matter yield (DMY) has emerged as a new target in rye breeding, which has been primarily driven by grain yield GY (Haffke et al. 2014). In contrast to GY, which in our breeding program is already tested at the first year of general combining ability testing (GCA-1), DMY is traditionally evaluated through destructive methods at later selection stages on a strongly reduced set of genotypes. Thus, lower selection gains can be expected due to the loss of important genetic variation during the breeding process.

Efficient indirect selection for dry matter yield (DMY) would, therefore, be needed to exploit the full genetic variation present at early selection stages. Plant height (PH) has been identified as an indirect selection target for enhanced DMY, but biomass-specific trials with a particular focus on lodging resistance were still recommended (Roux et al. 2010; Haffke et al. 2014). Genomic selection (GS) (Meuwissen et al. 2001) aims to indirectly select unphenotyped candidates based on a model trained in a reduced set of genotyped and phenotyped entries (training set, TRN). Genomic tools have been proposed to increase the efficiency of selection in hybrid rye breeding (Miedaner et al. 2019). For instance, GS has been recommended for enhanced prediction of grain yield in rye across breeding cycles (Auinger et al. 2016; Bernal-Vasquez et al. 2017). Another study in rye showed that, in terms of prediction accuracy, GS was preferred to marker-assisted selection (MAS) in intra-pool crosses not only for GY but also for PH and quality traits (i.e., starch and pentosan content, Wang et al. 2014).

The development of molecular techniques has increased the needs of reliable and cost-effective phenotypic information, representing a great challenge for the progress of plant-genetic studies (Araus and Cairns 2014; Montes et al. 2007). High-throughput phenotyping (HTP) has emerged as a suitable strategy for phenotyping thousands of new genotypes effectively and affordably based on reflectance information (Furbank and Tester 2011; White et al. 2012). Unmanned aerial vehicles (UAVs) such as polycopters outperform ground-based HTP platforms regarding working capacity while deriving high-resolution image data (Araus and Cairns 2014). So, they may represent a suitable approach for screening multi-environment field trials, exponentially increasing the amount of data available. In this context, a positive impact on practical plant breeding may be expected if reflectance data are associated with the target trait (Rutkoski et al. 2016). This would be of great interest, for instance, to enhance indirect estimation of DMY within a breeding population at first stage of GY trials, when a direct assessment of the trait by destructive measures would not be feasible, but aiming for a dual-purpose program with genotypes being superior for both DMY and GY.

Hyperspectral sensors deliver information of hundreds of wavelengths (hereafter referred as “bands”) at a nanometer-level resolution covering a broad spectral range (from 350 up to 2500 nm) that includes the visible spectrum (VS) and the infrared (IR) regions (Mahlein et al. 2012). This imaging technique is a promising tool for field phenotyping but presents additional computation efforts due to the increased data dimensionality (Fahlgren et al. 2015). To address this issue, several strategies have been proposed for integrating reflectance data into practical plant breeding. One approach is to summarize a few individual bands into vegetation indices (VIs; Xue and Su 2017; Galán et al. 2020). However, prediction accuracy of VIs was found to be lower than equations incorporating whole-spectrum data by ordinary least squares (OLS), partial least squares (PLS), and Bayesian shrinkage for GY prediction in maize (Aguate et al. 2017) and by Bayesian functional models in wheat (Montesinos-López et al. 2017).

In both studies, models were tested under *p* < *n* scenarios, where the number of predictors (*p*) was smaller than the population size (*n*). On the contrary, when *p* >  > *n* as in GS, regularization (penalized) models have shown to be suitable for incorporating thousands of predictors, including several unrelated to the trait of interest, or highly intercorrelated (Ogutu et al. 2012). A similar situation may be expected when analyzing hyperspectral data collected in several environments and on several dates. To reduce multicollinearity, increase prediction accuracy, minimize calculation time, and extract the most informative features, regularization methods such as the elastic net (Zou and Hastie 2005) or the least absolute shrinkage and selection operator (Lasso; Tibshirani 1996) are also preferred for facing high-dimensional spectral data (Liu and Li 2017).

Alternatively, Krause et al. (2019) found that deriving relationship matrices from hyperspectral data was a suitable approach to integrate whole-spectrum reflectance information into multi-kernel GS for predicting GY in wheat within multi-environment field trials. Multivariate models integrating correlated traits have demonstrated to be more precise than univariate models in GS (Jia and Jannink 2012). In wheat, for instance, GS prediction ability of GY was significantly enhanced by fitting traits derived from hyperspectral data (Sun et al. 2019; Rutkoski et al. 2016; Crain et al. 2018).

Similar to GS, models seeking the estimation of breeding values utilizing hyperspectral information also need phenotypic data (e.g., DMY) for model training. In our study case, the TRN size is economically highly relevant, since the acquisition of the phenotypic data requires to evaluate the candidates in GY-plots and DMY-plots separately under the conditions of a dual-purpose breeding program. The positive relationship between GS accuracy and TRN size is widely known (VanRaden et al. 2009). However, a broader TRN represents an increase in breeding costs. Thus, efficient breeding programs would benefit from reduced TRN while maintaining, or at least minimizing the loss of prediction accuracy. Approaches to enable the highest accuracy for a reduced TRN by integrating phenotypic and hyperspectral information to GS are, therefore, highly relevant for delivering high-yielding DMY varieties.

The aim of the present study was to test these approaches within the same breeding population by evaluating a set of 274 elite rye lines as a testcross series in multi-environment field trials on a phenotypic, genotypic, and hyperspectral level. In particular, the objectives were (1) to identify the most relevant spectral regions to DMY prediction in rye, (2) to integrate the different sources of information into multi-kernel and bivariate models for leveraging selection gain of DMY in rye, and (3) to compare prediction ability of models across different TRN sizes.

## Materials and methods

### Plant materials and field experiments

The plant materials and field experiments analyzed in the present study are described in detail in Galán et al. (2020). In short, a total of 264 recombinant inbred lines (RILs) of generation S_4_ (i.e., lines after continued self-fertilization of single plants for four consecutive years) were derived from ten diverse parental lines of the Petkus (seed parent) gene pool following a single round-robin design (Verhoeven et al. 2006). In practical plant breeding, these parental lines represent elite breeding material, since in contrast to a diverse panel of genetic resources, they were obtained after several selection cycles for line per se performance and general combining ability (GCA). Testcross seed was produced from the cross of these 264 RILs and their ten parental components with a single-cross tester from the opposite (pollinator) gene pool. The obtained 274 genotypes, thus, correspond to three-way hybrids, (A· B) × C. They were analyzed for their dry matter yield (DMY) and plant height (PH) in two trials with a size of 130 and 134 entries, respectively, laid out as resolvable incomplete block designs (α-lattice design) with two replicates. These field trials were grown adjacent to each other and conducted in 2017 and 2018 at each of four environmentally contrasting locations in Northern Germany (Suppl. Table 1), thus comprising eight environments (location–year combinations). Plots were harvested by a commercial plot chopper at late milk stage (BBCH 77; Meier 1997) to get the respective yield per plot as fresh matter yield (FMY, dt ha ^−1^). For DMY (dt ha ^−1^) determination, representative samples of about 1000 g were weighted from each plot and oven-dried at 110 °C till a constant weight was reached. Dry matter content (DMC) in percentage was determined from weight differences of the samples. DMY per plot was estimated as DMY = FMY × DMC/100. Also, PH (cm) was recorded at each plot.

### Hyperspectral data

Hyperspectral data consisting of 400 bands ranging from 410 to 993 nm were obtained in all environments and for all genotypes by an unmanned aerial vehicle (UAV; Camflight FX8HL, Sandnes, Norway) that was fitted with a hyperspectral camera (HySpex Mjolnir V-1240, Skedsmokorset, Norway) as described previously in detail Galán et al. (2020). Reflectance data were recorded after flowering (i.e., during the grain filling stage) at two flight dates in each environment, except for location Bernburg in 2017 (BBG 2017) where only one flight was conducted (Suppl. Table 1). On each flight date, the UAV quadcopter flew at about 25 m above plots, around solar noontime. Each plot was demarcated on the obtained images by a polygon, provided by digital geographic information system (GIS) field plans. Raw data were radiometrically calibrated (HySpex PostProcessor Version 1.2). This is a hyperspectral standard procedure (Adão et al. 2017) to convert the arbitrary digital numbers to values, which are proportional to the International System of Units (SI) unit W/sr nm m^2^ (HySpex Mjolnir‐1024 User’s Manual). Coefficients of incident sunlight were captured by placing a 70-by-150 cm wooden board painted gray in the center of the field and using it as a reference to account for different irradiance conditions at each data collection time. The chosen gray panel reflects 60% of incident sun light, minimizing the risk of oversaturation of the hyperspectral sensor under varying sunlight conditions. The spectrum from the gray reflection target was assumed to represent the maximal reflection for each wavelength derived from sunlight. Normalized hyperspectral data (NormHyp) were then estimated based on this spectrum according to the formula Normhyp = Hyperspectral reflectance/Gray panel spectrum. Further, hyperspectral imaging data were orthorectified and georeferenced via the PARGE Software (ReSe Applications LLC, Wil, Switzerland).

Finally, all data points per each wavelength within each polygon were spatially averaged, resulting in one spectrum per plot. Consequently, each plot contains a single value for each wavelength in the studied spectrum. A tabular data frame was constructed, including the computed reflectance values of all bands.

### Genotypic data

All 274 genotypes (264 RILs and their ten parental components) were genotyped with an Illumina INFINIUM chip with 9,963 single-nucleotide polymorphisms (SNPs) assays (KWS SAAT SE & Co. KG, Einbeck, Germany). The SNPs of this assay are partially overlapping with the 5 k-SNP assay of Martis et al. (2013) and the 600 k-SNP assay of Bauer et al. (2017), whereof 3017 markers have been previously mapped by Bauer et al. (2017). SNPs showing more than 10% of missing values or a minor allele frequency < 0.05 were excluded. Imputation of the missing values in the remaining set of SNPs was performed with Linkimpute (Money et al. 2015). After imputation, data were filtered again for low minor allele frequency (< 0.05). Thus, 6420 markers were retained for subsequent analyses.

### Phenotypic data analysis

Within and across environments, phenotypic data (i.e., DMY and PH) were analyzed by different mixed models to obtain variance components and BLUEs (best linear unbiased estimators) of genotypes for later use in prediction modeling.

A combined analysis across locations and years was conducted by applying the following mixed model:

1 $$\begin{aligned} \gamma &= G:L + Y \\&\quad+ L \cdot G + Y \cdot G + Y \cdot L + L \cdot Y \cdot G \\&\quad+{\text{ENV}} \cdot T + {\text{ENV}} \cdot T \cdot R + {\text{ENV}} \cdot T \cdot R \cdot B + e \end{aligned}$$

where $$\gamma$$ denotes the observed genotype performance, $$G$$ the genotypes,$$ L$$ the locations, $$Y$$ the years, $$T$$ the trials within environments $${\text{ ENV}}$$ (equivalent to year–location combinations), $$R$$ the replicates within trials, $$B$$ the blocks within replicates, and $$e$$ the error associated with the observation $$\gamma$$. Error, trial, block, and replicate variances were assumed heterogeneous among environments. In model (1), the dot operator (·) specifies crossed effects (A·B) and fixed and random terms are separated by a colon (:), with fixed terms appearing first (Piepho et al. 2003). Variance components and pairwise variances of genotype mean (BLUEs) differences (needed for heritability estimation) were estimated by restricted maximum likelihood (REML) for all random effects in model (1). This also holds for estimation of the genotypic variance ($$\sigma_{g }^{2}$$), which required an additional analysis fitting the above model with random genotypic effects. Significance of variance component estimates was tested by model comparisons using likelihood ratio tests (Stram and Lee 1994).

BLUEs of genotypes were also analyzed within environments by the following mixed model:

2 $$ \gamma = G:T + T \cdot R + T \cdot R \cdot B + e $$

This model (2) differs from the first model (1) only in dropping the year and location main effects and corresponding interactions with genotypes. Variance components for single environments were estimated as described previously for model (1). Phenotypic outliers were tested for DMY and PH based on the Bonferroni–Holm test (method “M4r”; Bernal-Vasquez et al. 2016). Plots flagged as outliers were excluded from the analysis. Hyperspectral information was excluded from plots flagged as an outlier for DMY.

### Three-stage analysis for DMY prediction

To reduce computing cost, prediction ability of DMY based on different information sources was conducted by a three-stage procedure (Piepho et al. 2012), where in the first two stages, hyperspectral data were analyzed across dates and environments to obtain BLUEs per genotype, which were then incorporated into DMY prediction models in the last stage.

#### First-stage models

In the first stage, hyperspectral bands were adjusted across dates per environment according to the model

3 $$\begin{aligned} \gamma& = G:D + D \cdot G \\&\quad+ T + T \cdot R + T \cdot R \cdot B \\&\quad+ D \cdot T + D \cdot T \cdot R + D \cdot T \cdot R \cdot B + e \end{aligned} $$

where $\gamma$ is the observed band value, $G$ the genotypes, $D$ the measurement dates, $T$ the trials, $R$ the replicates within trials, $B$ the blocks within replicates, and $e$ the error associated with the observation $\gamma$. Errors of different measurement dates on the same plot are correlated; therefore, a correlation structure (“Compound Symmetry”) was assumed for *e* as described in Piepho et al. (2004). This model was used here because there were only two measurement dates per environment. The random effects for trials, replicates, and blocks also imply a compound symmetry variance–covariance structure for repeated observations on these units. For BLUEs estimation, all factors included in model (1) except $G$ were considered as random. For single bands in each flight date (“first” and “second”), the random effects of the date, including the corresponding interaction terms, were excluded from model (3). To allow a fair comparison between across and within flight dates, data collected in BBG (2017), where only one flight was conducted, were included in both single-date and across-dates analyses.

#### Second-stage models

In the second stage, variance components and BLUEs per genotypes were estimated across environments following the model

4 $$ \gamma = G:{\text{ENV}} + G\cdot{\text{ENV}} + e $$

where $\gamma$ is the adjusted genotype mean (BLUEs) from the first stage for the band value, $G$ and ${\text{ENV}}$ denote genotypes and environments, respectively, and $e$ is the error associated with the observation $\gamma$. When adjusted means from the first stage are forwarded to second-stage models, the incorporation of a weighting method is preferable (Möhring and Piepho 2009). Means were therefore weighted by the diagonal elements of the inverse of their variance–covariance matrix calculated in the first stage as proposed by Smith et al. (2001). For hyperspectral data, estimates of variance components, pairwise variances of genotype mean differences (BLUEs) as well as significance tests of variance components were computed as for the phenotypic data. The syntax of models (1), (2), (3), and (4) is also compatible.

At this stage, heritability ($h^{2}$) was estimated for DMY, PH, and each band for single and for combined flight dates across environments as (Piepho and Möhring 2007)

5 $$ h^{2} = \frac{{\sigma_{g }^{2} }}{{\sigma_{g}^{2} + \frac{{\overline{v}}}{2}}} $$

where $\overline{v}$ is the mean variance of a difference between two adjusted genotype means (BLUEs) derived from model (1) or from model (4) for phenotypic and hyperspectral data, respectively. All statistical analyses were performed within the R-environment v. 3.4.4 (R Core Team 2018). BLUEs of genotypes were calculated with the software package ASReml-R v. 3.0 (Gilmour et al. 2009).

#### Third-stage models

In the third stage, the obtained phenotypic and hyperspectral BLUEs from model (1 or 2) and (3 or 4), respectively, were used for fitting several models (described in Table 1) for predicting DMY, including genetic, hyperspectral, and phenotypic data. A weighting method was applied also on this stage as described before, with weights derived from models (1) or (2).

**Table 1:** Overview over the models used

Model	Integrated variables
Single-kernel models	
GBLUP	Genotypic data
HBLUP	Hyperspectral data
Multi-kernel model	
G + H	Genotypic + hyperspectral data
Bivariate models	
Bivariate_G	Genotypic data + plant height
Bivariate_H	Hyperspectral data + plant height
Bivariate_G + H	Genotypic + hyperspectral data + plant height

The predictive power of these models was assessed in two different scenarios: (S1) across the series of eight environments by cross-validation (CV) and (S2) by fitting prediction models with data collected on a variable number of environments (*E* = 1,2,…,7), while one environment not included in E was used for model validation. Coefficients of phenotypic correlation $r$ (Pearson’s coefficients of correlation) between DMY and all other traits were calculated from the BLUEs of genotypes from model (1) or (2) for prediction scenarios S1 or S2, respectively.

Third-stage models were single-kernel and multi-kernel prediction models, providing best linear unbiased predictions (BLUP) of genotypic effects of DMY, which differ in the information used to model the random genotypic effect. Single-kernel prediction models were fitted with genetic (genomic BLUP, GBLUP) or hyperspectral (hyperspectral BLUP, HBLUP) information with *n* = 274 individuals, based on *m* SNP markers or *b* bands, respectively. Thus, genomic estimated breeding values (GEBVs) were derived from the GBLUP model, whereas hyperspectral estimated breeding values (HEBVs) were obtained from the HBLUP model.

The two models were defined as

6 $$ \rm{GBLUP}:\varvec{\it{y}} = \mu\varvec{1}_{{\varvec{n}}} + {\varvec{\it{g}}}_{{\varvec{K}}} + {\varvec{e}}, $$

7 $$ \rm{HBLUP}:\varvec{\it{y}} = \mu\varvec{1} _{{\varvec{n}}} + {\varvec{\it{g}}}_{{\varvec{H}}} + {\varvec{e}}, $$

where ${\varvec{y}}$ is the *n*-dimensional vector of BLUEs of DMY obtained from model (1) or model (2) for prediction scenarios S1 or S2, respectively, $\mu$ is the overall mean, $1_{{\varvec{n}}}$ an *n*-dimensional vector of ones, ${\varvec{g}}_{{\varvec{K}}}$ and ${\varvec{g}}_{{\varvec{H}}}$ are *n*-dimensional vectors of random genotypic effects, and ${\varvec{e}}$ is the *n*-dimensional vector of residuals. The vector of residuals $e$ associated with ${\varvec{y}}$ was assumed as normally distributed with zero mean and variance **R** [${\varvec{e}}$ ~ N (0, **R**)]. **R** is defined as a diagonal matrix with diagonal elements equivalent to the inverses of the diagonal elements of inverse of the original variance–covariance matrix of the means adjusted on the second stage of this analysis (Smith et al. 2001). When means adjusted in the second stage are forwarded to third-stage models, the incorporation of a weighting method was performed as described before.

For GBLUP, the random genetic values were estimated as ${\varvec{g}}_{{\varvec{K}}}$ ~ N (0, **G **$\sigma_{g}^{2}$) where $\sigma_{g}^{2}$ is the genetic variance and **G** the genomic additive relationship matrix (Habier et al. 2013). For estimating genotypic values based on hyperspectral data, the random genetic values in model 7 were calculated as ${\varvec{g}}_{{\varvec{H}}}$ ~ N (0, **H** $\sigma_{b}^{2}$) where $\sigma_{b}^{2}$ is the hyperspectral band variance and **H** a hyperspectral reflectance-based relationship matrix.

**G** was estimated with the synbreed package (Wimmer et al. 2012) in R following the first method of VanRaden (VanRaden 2008) as ${\text{G}} = \frac{{{\text{ZZ}}^{\prime } }}{{2 \sum p_{i } (1 - p_{i } )}}$, where Z = M-P, M is the *n* × *m* marker matrix of alleles coded as 0 (A_1_A_1_), 1 (A_1_A_2_), or 2 (A_2_A_2_) for the *n*th individual at the *m*th SNP position, *P* contains a *n* × *m* matrix of allele frequencies multiplied by 2, $p_{i }$ is the allele frequency of the *i*th allele.

**H** was also calculated for the *n* = 274 genotypes by incorporating the BLUEs for each band derived from model (4) or (3) for prediction scenarios S1 and S2, respectively. These matrices were of the form ${\text{H}} = {\text{DD}}^{\prime }$, where $D$ is a *n* × *b* hyperspectral matrix of the standardized BLUEs of the bands. Standardization was done by subtracting the arithmetic mean and dividing by the standard deviation of all BLUEs. For **H** estimation, different numbers of bands were considered: **H**_**all**_ is derived from the total number of bands available (*b* = 400), whereas **H**_**vsel**_ (*b* = 32) and **H**_**h2**_ (*b* = 216) are based on a reduced set of bands. Bands included in **H**_**vsel**_ were selected as described in the next sections, while **H**_**h2**_ is based only on bands with $h^{2}$ larger than the mean value observed for all bands ($h^{2}$ > 0.72).

Finally, a multi-kernel prediction model combining genetic and hyperspectral information was fitted:

8 $$ {\varvec{y}} = \mu 1_{{\varvec{n}}} + {\varvec{g}}_{{\varvec{K}}} + {\varvec{g}}_{{\varvec{H}}} + {\varvec{e}}, $$

where all factors listed are defined as above in models (6) and (7). The random vectors ${\varvec{g}}_{{\varvec{K}}}$ and ${\varvec{g}}_{{\varvec{H}}}$ in (8) are considered as independent of each other and normally distributed. Here, the **H** matrix assumes the form of **H**_**vsel**_. For exploring the benefits of incorporating PH as a predictor, model (9) was extended to a bivariate model (Bivariate_G + H) as

9 $$ \left[ {\begin{array}{*{20}c} {{\varvec{y}}_{1} } \\ {{\varvec{y}}_{2} } \\ \end{array} } \right] = \left[ {\begin{array}{*{20}c} {1_{{\varvec{n}}} }  {0_{{\varvec{n}}} } \\ {0_{{\varvec{n}}} }  {1_{{\varvec{n}}} } \\ \end{array} } \right]\left[ {\begin{array}{*{20}c} {\mu_{1} } \\ {\mu_{2} } \\ \end{array} } \right] + \left[ {\begin{array}{*{20}c} {{\varvec{g}}_{\varvec{K1}} } \\ {{\varvec{g}}_{\varvec{K2}} } \\ \end{array} } \right] + \left[ {\begin{array}{*{20}c} {{\varvec{g}}_{\varvec{H1}} } \\ {{\varvec{g}}_{\varvec{H2}} } \\ \end{array} } \right] + \left[ {\begin{array}{*{20}c} {{\varvec{e}}_{\varvec{1}} } \\ {{\varvec{e}}_{\varvec{2}} } \\ \end{array} } \right] $$

where ${\varvec{y}}_{1}$ is a vector of BLUEs for DMY, ${\varvec{y}}_{2}$ is a vector of BLUEs for PH, with ${\varvec{y}}_{1}$ and ${\varvec{y}}_{2}$ incorporating BLUEs derived from model (1) for prediction scenario S1, $\mu_{1}$ is the overall mean for DMY, $\mu_{2}$ is the overall mean for PH, $g_{k1}$ and $g_{H1}$ are *n*-dimensional vectors of random effects for DMY, ${\varvec{g}}_{\varvec{K2}}$ and ${\varvec{g}}_{\varvec{H2}}$ are *n*-dimensional vectors of random effects for PH, $e_{1}$ is the *n*-dimensional vector of residuals for DMY, and $e_{2}$ is the *n*-dimensional vector of residuals for PH. The random vectors are considered as independent of each other and normally distributed according to $$\left[ {\begin{array}{*{20}c} {{\varvec{g}}_{\varvec{K1}} } \\ {{\varvec{g}}_{\varvec{K2}} } \\ \end{array} } \right]$$ ~ N(**0**, **C**_**K**_ ⊗ **G**),$$\user2{ }\left[ {\begin{array}{*{20}c} {{\varvec{g}}_{\varvec{H1}} } \\ {{\varvec{g}}_{\varvec{H2}} } \\ \end{array} } \right]$$ ~ N(**0**, **C**_**H**_ ⊗ **H**), and $$\left[ {\begin{array}{*{20}c} {{\varvec{e}}_{1} } \\ {{\varvec{e}}_{2} } \\ \end{array} } \right]$$ ~ N(0, **R** ⊗ **I**), where **G** is defined as in model (4), ⊗ is the Kronecker product (direct product) operator, **C**_**K**_ and **C**_**H**_ are the 2 × 2 variance–covariance matrices for the breeding values of the two traits**, H** is defined as in model (7) and adopts the form of **H**_**vsel**_, **I** is an identity matrix, and **R** is the residual variance–covariance matrix for DMY and PH. The covariance matrices **C**_**K**_**, C**_**H**_**,** and **R** were considered unstructured. At this stage, model (9) was fitted without a weighting method to reduce computing costs. Bivariate_G + H aims to predict DMY based on PH as well as hyperspectral and genetic data. For addressing the impact of PH on the predictive power of bivariate models based only on hyperspectral (Bivariate_H) or genetic (Bivariate_G) data, two additional bivariate models were analyzed. These two models are a reduced version of model (9). For models Bivariate_H and Bivariate_G, the terms.

$$\left[ {\begin{array}{*{20}c} {{\varvec{g}}_{\varvec{K1}} } \\ {{\varvec{g}}_{\varvec{K2}} } \\ \end{array} } \right]$$ or $$\left[ {\begin{array}{*{20}c} {{\varvec{g}}_{\varvec{H1}} } \\ {{\varvec{g}}_{\varvec{H2}} } \\ \end{array} } \right]$$ were dropped, respectively. All three-stage prediction models were fit using the R package "sommer" (Covarrubias-Pazaran 2016).

#### Feature selection for the hyperspectral data

Multicollinearity in regression equations is expected when numerous highly intercorrelated hyperspectral variables are incorporated (Dunagan et al. 2007). To overcome this, two variable selection methods were used and implemented in the GlmNet R package (Friedman et al. 2010). Since weighted and unweighted variable selection procedures yielded similar results, we performed the following methods without the incorporation of a weighting factor.

The least absolute shrinkage and selection operator (Lasso; Tibshirani 1996) is a well-known and powerful regression method for regularization and variable selection for minimizing the prediction error. Applying the $l1$ penalty sets some of the regression coefficients to zero, while others are shrunk toward zero yielding a sparse solution. The Lasso should, however, be used with care in the case of sets of highly correlated variables since it tends to arbitrarily select one variable and overlook the rest (Friedman et al. 2010).

The elastic net (EN; Zou and Hastie 2005) was developed to overcome the restrictions of Lasso. It combines both $l1$ (Lasso) and $l2$ (Ridge Regression, Hoerl and Kennard 1970) penalization terms to obtain a more stable solution to highly correlated predictors.

The estimators ($\hat{\beta }$) for Lasso and EN can be calculated from the following penalized equation (Wimmer et al. 2013):

10 $$ \varvec{\hat{\beta }} =_{{ {\varvec{\beta}}}}^{\rm{arg\,min}} \Vert{}{\varvec{\gamma}} - \varvec{{X\beta }}\,\Vert{}_{2}^{2} + \rm{Pen}\left( \beta \right) $$

where ${\varvec{\gamma}}$ is defined as in model (4), ${\varvec{X}}$ is a *n* × *b* matrix of bands; ${\varvec{\beta}}$ is the vector of the regression coefficients of the bands; ${\text{Pen}}\left( {\varvec{\beta}} \right)$ is the penalization term, which is defined by the quadratic $l2$ norm for RR as ${\text{Pen}}\left( {\varvec{\beta}} \right) = \lambda\, \Vert{}\,\varvec{{\beta }}\,\Vert{}_{2}^{2}  = \lambda \mathop \sum \nolimits_{{j = 1}}^{p} \varvec{{\beta }}_{j}^{2}$ , by the $l1$ norm for Lasso with $\rm{Pen}\left( \varvec{{\beta }} \right) = \lambda \,\Vert{}\,\varvec{{\beta }}\,\Vert{}_{1}  = \lambda \mathop \sum \nolimits_{{j = 1}}^{p} \left| {\varvec{{\beta }}_{j} } \right|$, and for EN by combining both as $\rm{Pen}\left( {\varvec{\beta}} \right) = \lambda_{1} {\varvec{\beta}}_{1} + \lambda_{2} {\varvec{\beta}}_{2}^{2}$. For EN, the procedure can be described as a penalized least square method with $\alpha = \frac{{\lambda_{2} }}{{\lambda_{1} + \lambda_{2} }}$; thus, Eq. (10) is equivalent to the optimization problem $\varvec{\hat{\beta }} =_{{ {\varvec{\beta}}}}^{\rm{arg\,min}} \Vert{}{\varvec{\gamma}} - \varvec{{X\beta }}\,\Vert{}_{2}^{2}$, subject to $P_{\alpha } \left( {\varvec{\beta}} \right) = \left( {1 - \alpha } \right){\varvec{\beta}}_{1} + \alpha {\varvec{\beta}}_{2}^{2} \le s$ for some $s$.

For fitting and comparing the Lasso and EN models, the optimal values for the tuning parameter ($\lambda \ge 0$), which control the degree of shrinkage of the estimator, were obtained by tenfold cross-validation with the function cv.glmnet of the GlmNet R package (Friedman et al. 2010) with default settings. In addition, for the defined optimal $\lambda$, the best value for $\alpha$ for the EN was estimated outside the GlmNet package by a tenfold cross-validation.

### Validation of variable selection procedures and proposed prediction models

In the present study, two prediction scenarios were considered, namely S1 and S2. A fivefold cross-validation (CV) was used to assess the predictive ability of models in S1, where models were fitted to fourfold (~ 219 genotypes), and model error was estimated when predicting the remaining validation fold (~ 55 genotypes). This was conducted for all five possible validation folds, and the obtained estimates of prediction error were combined. This procedure was repeated 100 times (i.e., 500 cross-validations), each repetition with a random composition of folds to assess CV error.

To investigate the effect of the TRN size on the prediction ability of all models in scenario S1, TRN was sampled according to a defined size (i.e., 55, 110, 165, or 220 individuals) and the validation set (VAL) consisted on the remaining genotypes. As described before, models were fitted to the TRN and model error was determined when predicting the VAL. This process, including the random sampling of the TRN, was repeated 500 times. For the larger TRN size, the prediction models were further evaluated. This procedure consisted of extracting, at each CV iteration, the predicted best yielding genotypes ranked above certain thresholds (10, 20, 30, and 40%). Then, the performance of the selected fraction was assessed in terms of its observed DMY and PH according to the BLUEs derived from model (1). Finally, the prediction ability of each model for each selected fraction was estimated as described below (see suppl. Table S4).

In scenario S2, HBLUP fitted with **H**_**all**_ was tested across all possible combinations between E and validation environments. Also, the environmental distinctiveness was assessed by the discriminant analysis of principal components DAPC (Jombart et al. 2010) using the R package adegenet (Jombart 2008) based on hyperspectral BLUEs derived from model (3).

For all validation approaches, prediction ability for DMY was assessed as the correlation $r$ between estimated breeding values and the observed BLUEs derived from model (1) for S1 and from model (2) for S2. Predictive abilities of bivariate models were estimated based on PH, hyperspectral, and genetic data (for Bivariate_H and Bivariate_G, only the corresponding data were included), whereas DMY was additionally used only for model training. Mean prediction abilities were compared according to Tukey’s honestly significant difference (HSD) test (*p* < 0.01) with the R package multcomp (Hothorn et al. 2016). For Lasso and EN, each predictor (band), whose regression coefficient was not set to zero ($\hat{\beta } \ne 0$), was extracted and saved in a tabular form. Across variable selection runs, bands retaining > 40% of the time were considered as selected (recovery rate). The regularization method with the highest prediction ability based on the smallest number of selected bands was considered as the best procedure for reducing multicollinearity in the hyperspectral data. For **H**_**vsel**_ estimation, selected bands derived from the best regularization scheme were used.

## Results

In the present study, hyperspectral data were collected by two different flights performed after the heading stage, which were analyzed both individually and jointly. For all the issues under analysis, similar trends with no major contradictions could be observed, regardless of the number of flights considered. Therefore, the following sections are based on the joint analysis of both flight dates. The main results of the adjustment of individual flights can be found in the supplementary files (Suppl. Fig. S2, Suppl. Fig. S5, Suppl. Table S3).

### Heritability and correlation estimates

**Fig. 1:**
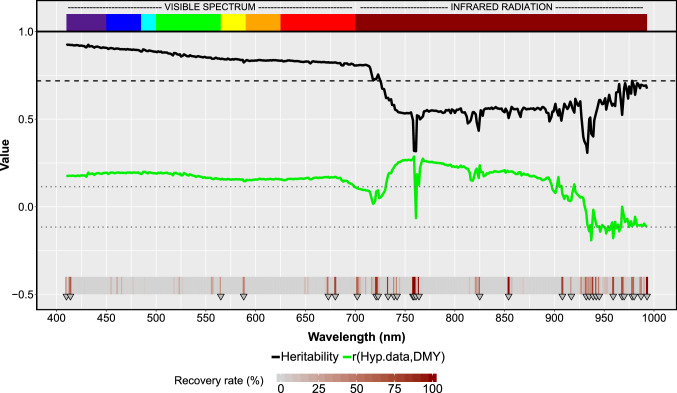
Heritability estimates (black line) for the hyperspectral bands, phenotypic correlations (*r*, green line) between hyperspectral bands and dry matter yield, and recovery rate (%) of hyperspectral bands after the least absolute shrinkage and selection operator (Lasso, gray-red heatmap) for 274 winter rye hybrids assessed in eight environments and two flight dates. The mean heritability across all wavelengths is denoted by the dashed black line. Correlation values $\ge \left| {0.12} \right|$ are significant (*p* < 0.05) as shown by the gray dotted lines. Selected hyperspectral bands (recovery rate > 40%) are indicated by the gray triangles (Lasso variable selection)

**Fig. 2:**
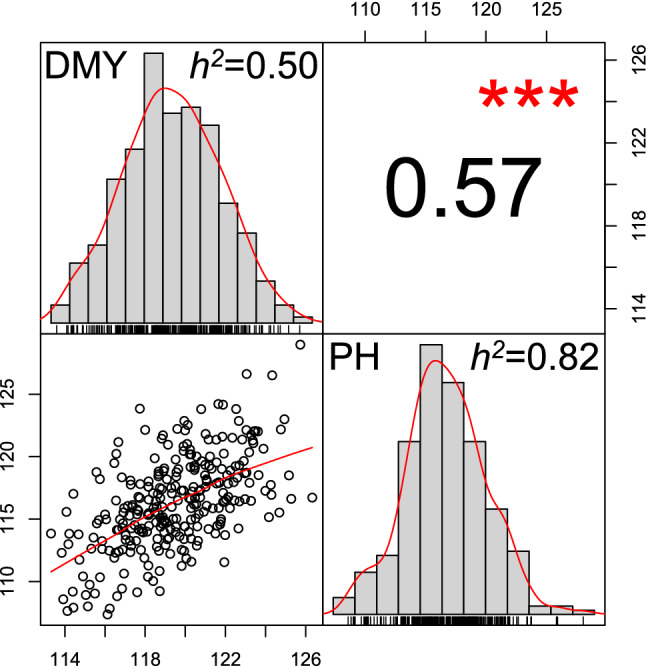
Histograms of dry matter yield (DMY) and plant height (PH) as well as the phenotypic correlation between both traits, determined for 274 winter rye hybrids assessed in eight environments. $h^{2}$ shows the heritability estimates of both traits. ***Significant at the 0.001 probability level

Across eight environments and two flight dates, the mean heritability of the reflectance data was moderate ($h^{2}$ = 0.72, Fig. 1). VS bands had mostly higher estimates than those from the IR. Generally, $h^{2}$ decreased in the VS with higher wavelength, while the opposite was observed for the IR. Estimates were highly variable among the whole spectrum (from 0.31 to 0.92), especially in the red edge region (~ 720–750 nm), wherein about 30 nm, $h^{2}$ dropped from 0.73 (720 nm) to 0.32 (761 nm). Also, DMY and PH were analyzed in the present study and showed moderate ($h^{2}$ = 0.50 for DMY) to high ($h^{2}$ = 0.82 for PH) estimates (Fig. 2).

The magnitudes of the correlations involving DMY were higher for PH ($r$ = 0.57, *p* < 0.001, Fig. 2) than for each of the 400 bands. Between DMY and the hyperspectral data, $r$ ranged from −0.19 for bands around 930 to 0.29 nm for bands around 750 nm. Estimates $\ge \left| {0.12} \right|$ were significant at the 5% probability level. The mean correlation among bands in the VS was slightly higher than the observed for the IR (0.17 and 0.11, respectively). On the other hand, bands were highly intercorrelated. Bands within the VS as well as within the IR were highly positively intercorrelated (Suppl. Fig. S1). In contrast, correlations between both regions were highly negative. Interestingly, $r$ was very low between a small group of bands from the red edge region and the rest of the spectrum.

### Feature selection for the hyperspectral reflectance data

The two regularization methods (Lasso and EN) applied to the hyperspectral data performed similarly when predicting DMY (*r* = 0.54, Suppl. Fig. S3). However, they were based on a different number of selected variables (Suppl. Fig. S4). From the total 400 available bands, only 32 (~ 8%, Suppl. Table S2) and 54 (~ 13%) bands were selected by Lasso and EN, respectively. EN selected more bands than Lasso; however, all chosen bands by Lasso were also included in the EN selection (Suppl. Fig. S4).Thus, Lasso emerges as the procedure of choice for the present study because it yielded the same predictive power as EN but is based on a simpler prediction model. From the 32 selected bands by Lasso, 26 corresponded to the IR and only six to the VS (Fig. 1). These 26 bands were mostly located at both ends of IR (700–780 nm and 925–993 nm). Selected bands for the individual flight dates can be also found in Suppl. Fig. S4.

### Prediction abilities of models

**Fig. 3:**
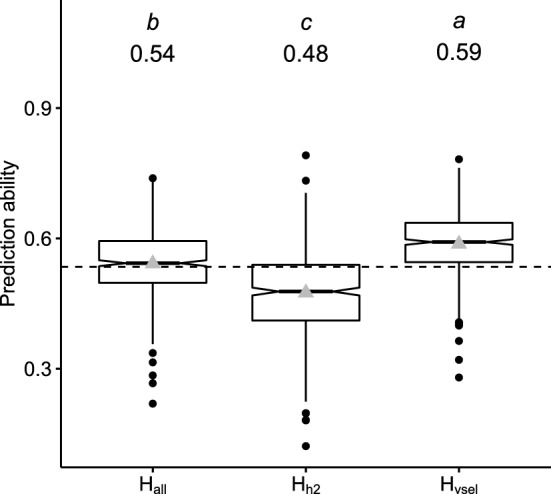
Prediction ability for dry matter yield of hyperspectral best linear unbiased predictor model (HBLUP) based on different **H** relationship matrices, including all available 400 bands (**H**_**all**_), bands with heritability > 0.72, **(H**_**h2**_**)**, and only selected bands by Lasso (**H**_**vsel**_) for 274 winter rye hybrids. Mean values are shown above each box plot and by gray triangles and are significantly different when headed by no letter in common (Tukey’s honestly significant difference test; *α* = 0.01%). The dashed line shows the mean value across models

Two key factors largely affecting the accuracy of prediction models based on reflectance data were investigated, namely the composition of the **H** relationship matrix and the TRN size. For addressing the first factor affecting HBLUP predictive power, three HBLUP models based on dissimilar **H** relationship matrices (**H**_**all**_, **H**_**h2**_, and **H**_**vsel**_) were evaluated across the series of environments (Fig. 3, Suppl. Fig. S5). Thus, models differed in their number and composition of incorporated bands. In terms of prediction ability, the composition of **H** was highly relevant. Across environments, models incorporating all available bands ($r$ = 0.54) or only bands selected by Lasso ($r$ = 0.59) were considerably more accurate than models based only on bands with heritabilities > 0.72 ($r$ = 0.48). For scenario S1, HBLUP models based on **H**_**all**_ and **H**_**h2**_ were therefore discarded and hereafter HBLUP models are all based on **H**_**vsel**_.

For addressing the second factor (i.e., the TRN size), the performance of genotypes in scenarios S1 and S2 was predicted based on TRN of increased size. In S1, the prediction ability of proposed single-kernel, multi-kernel, and bivariate models (Table 1) was assessed with variable TRN sizes across environments (Table 2). The TRN sizes evaluated ranged from 55 (~ 20%) to 220 individuals (~ 80%). In general, the larger the TRN size, the higher the prediction ability of all models, and the lower their variance. The Bivariate_G + H model showed the highest prediction ability across TRN sizes, followed by the Bivariate_G model, the multi-kernel prediction model, the Bivariate_H model, and the single-kernel models (HBLUP and GBLUP). On the other hand, Bivariate_G + H was associated with the highest variability in reduced TRN sizes. This is in particular observable in Suppl. Fig. S6, where this model was compared with single-kernel and multi-kernel models. The model Bivariate_G + H estimates breeding values of genotypes based on PH, genotypic, and hyperspectral data, while the multi-kernel model does not include PH. For the smaller TRN size, the former showed a predictive ability of 0.56, while the latter yielded a predictive ability of 0.46. For the largest TRN size, their prediction ability was 0.75 and 0.71, respectively. Interestingly, across different selection intensities, the three bivariate models consistently selected the taller genotypes, which were not always associated with the highest DMY. In contrast, the multi-kernel model selected relatively shorter genotypes with an acceptable yield (Supp. Table S4). Both single-kernel models performed similarly with larger TRN sizes. For example, for TRN size of 80%, $r$ was close to 0.60. On the other hand, HBLUP was more predictively accurate than GBLUP based on smaller TRN sizes. For a TRN size of 55, HBLUP ($r$ = 0.42) surpassed GBLUP ($r$ = 0.32) by about 25%. A comparison among prediction models based on single flight data under validation scenario S1 is shown in Suppl. Table S3.

**Table 2:** Mean prediction abilities and standard errors for dry matter yield of six models across different training set sizes for 274 winter rye hybrids assessed in eight environments across two flight dates

Model^**a**^	Training set size^**b**^			
	20 (%)	40 (%)	60 (%)	80 (%)
GBLUP	0.32^a^ ± 0.002	0.44^a^ ± 0.002	0.54^a^ ± 0.002	0.60^a^ ± 0.003
HBLUP	0.42^b^ ± 0.004	0.51^b^ ± 0.002	0.56^b^ ± 0.002	0.59^a^ ± 0.003
G + H	0.46^c^ ± 0.003	0.59^d^ ± 0.002	0.66^d^ ± 0.002	0.71^d^ ± 0.003
Bivariate_G	0.54^e^ ± 0.004	0.61^e^ ± 0.002	0.66^d^ ± 0.002	0.69^c^ ± 0.003
Bivariate_H	0.50^d^ ± 0.005	0.55^c^ ± 0.004	0.60^c^ ± 0.002	0.62^b^ ± 0.003
Bivariate_G + H	0.56^f^ ± 0.007	0.65^f^ ± 0.004	0.71^e^ ± 0.003	0.75^e^ ± 0.002

^**a**^See Table 1 for more information about the listed models

^**b**^Within a column, means with no letter in common are significantly different (Tukey’s honestly significant difference test; *α* = 0.01%)

**Fig. 4:**
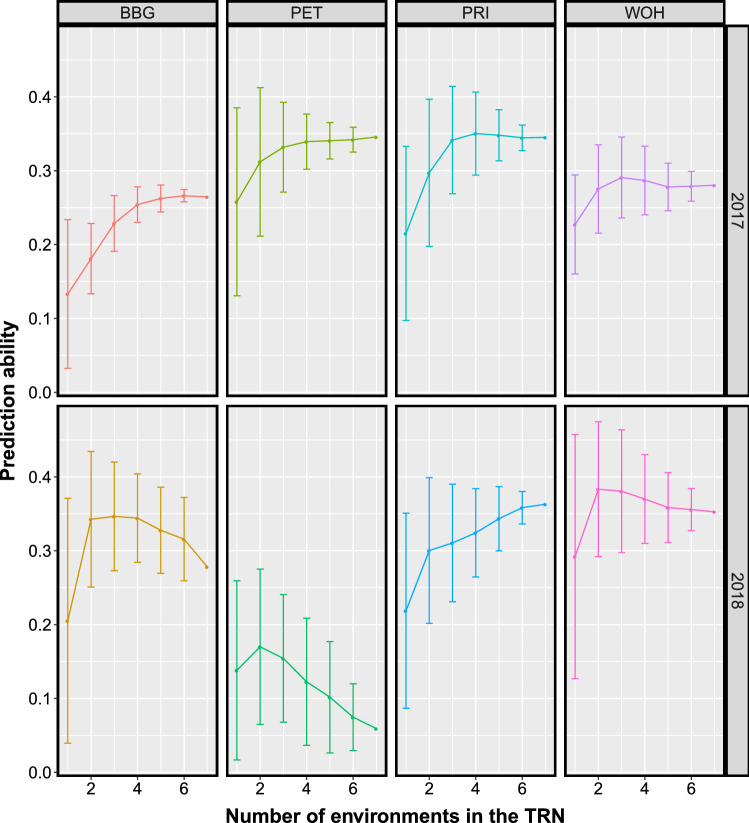
Prediction ability for dry matter yield of the hyperspectral best linear unbiased predictor model (HBLUP) on each environment with increased number of environments included in the training set (TRN). Models were tested under validation scenario S2

In S2, predictions were based on HBLUP models fitted with all bands (**H**_**all**_) collected in a variable number of environments. These environments were highly diverse according to a discriminant analysis (DAPC) based on reflectance data (Suppl. Fig. S7). Locations from the same year were mostly clustered together. The PET (2018) environment, on the other hand, was distinct to both clusters. Overall, the prediction of DMY in individual environments was improved by increased TRN size (Fig. 4). Thus, the higher the number of environments included in the TRN, the more accurate the prediction. However, the prediction ability was highly variable across environments. For TRN including only one environment, WOH (2018) showed the highest prediction ability ($r$ = 0.29), while BBG (2017) showed the lowest ($r$ = 0.13). When seven environments were considered as TRN, DMY had the highest prediction ability in PRI (2018) ($r$ = 0.36), while it had the smallest in PET (2018) ($r$ = 0.06).

## Discussion

The high versatility of rye as a dual-use crop (Miedaner and Laidig 2019) contrasts with traditional breeding programs, which are mainly driven by GY (Geiger and Miedaner 2009). Thus, the improvement of DMY is often pushed into later selection stages. To overcome this situation, an effective indirect estimation of DMY based on data collected on GY plots would be needed. Thus, in the present study, single-kernel, multi-kernel, and bivariate models based on different information sources collected within the same breeding population were compared regarding their DMY prediction ability across different validation approaches.

### Impact of heritability estimates of bands on HBLUP models

Across the spectrum, the magnitude and variability of $h^{2}$ estimates were higher than those of the correlation ($r$) between bands and DMY for combined (Fig. 1) and single flight dates (Suppl. Fig. S2). Highly variable $h^{2}$ for bands were also reported in wheat (Krause et al. 2019; Montesinos-López et al. 2017). We observed that $h^{2}$ and $r$ showed the greatest variability and the lower values within the IR. HBLUP models exploiting all available bands were substantially more precise than those fitted only with highly heritable bands ($h^{2}$ > 0.72). This seems counterintuitive from a breeding perspective since, according to quantitative-genetic theory (Falconer and Mackay 1996), highly heritable secondary traits correlated with the feature of interest are preferred for indirect selection of the target trait. A possible explanation of the low performance observed by models based on bands with $h^{2}$ > 0.72 is that the proposed threshold excluded almost all the bands belonging to IR. Despite their relatively lower mean $r$ with DMY, based on our results, this spectral region still captures information closely related to DMY, since bands around 750 nm had the highest correlation with DMY (Fig. 1). The magnitudes of these correlations were rather low but significant (< 0.29; *p* < 0.05) and are comparable to those stated for biomass in wheat by Hansen and Schjoerring (2003).Thus, the exclusion of bands from the IR because of their relatively lower $h^{2}$ deteriorated the predictive power of HBLUP models. This is in agreement with Montesinos-López et al. (2017), who found that GY prediction in wheat was not improved by removing bands with lower $h^{2}$.

### Reduction in the dimensionality of hyperspectral data

High-throughput phenotyping is a promising tool for overcoming the phenotyping bottleneck in modern plant breeding (Araus and Cairns 2014). On the one hand, the use of hyperspectral sensors can substantially increase the amount of data available for dissecting the genetics behind the trait of interest. On the other hand, the application of this technology on multi-environmental trials is computationally and economically challenging.

The exploitation of a vast amount of hyperspectral data should be performed with caution, since the combination of a large number of predictors, each with small effects, can negatively influence the accuracy of regression models (Ogutu et al. 2012). The high multicollinearity found among contiguous bands (Suppl. Fig S1) suggests that performing variable selection could be beneficial. In this context, Lasso was a valuable tool for reducing the number of predictors incorporated into the HBLUP model. Also, with the constant development of high-resolution HTP sensors, the utility of feature selection procedures may be increased in proportion to the incorporation of broader spectral regions.

### Informativeness of the VS and IR spectral regions

Use of HTP based on hyperspectral sensors can be time-consuming and resource-intensive although recent substantial improvements have occurred. Considerable overlaps were observed among specific bands highlighted by Lasso and EN in single and combined flight dates (Suppl. Fig. S4). Therefore, the reflectance data from these specific wavelengths may be of great interest to practical plant breeders. Redirecting computational costs toward these selected regions could reduce the efforts in data management. By this, the superiority of hyperspectral sensors in terms of data collection and calibration compared to cheaper devices covering fewer reflectance regions (e.g., RGB cameras, Araus and Cairns 2014) may be fully exploited in a less resource-demanding manner.

In the present study, bands across the whole spectrum showed a significant correlation with DMY, with the IR displaying the highest correlation estimates (Fig. 1). Also, when the IR was excluded, the prediction ability of HBLUP substantially dropped as discussed above. The variable selection procedures applied have highlighted single bands located in the VS and the IR as highly informative for DMY prediction (Fig. 1, Suppl. Table S2, Suppl. Fig. S4). Nevertheless, the majority of the selected bands were located within the IR. These findings suggest that all spectral regions contain information potentially useful for DMY prediction; however, IR may be more informative than of VS.

These findings also indicate that a reduction in predictive power is expected if spectral fingerprints of genotypes are based on a reduced number of spectral regions. This is consistent with literature highlighting the importance of the VS and the IR in assessing essential plant parameters. The behavior of plants exposed to visible light has been widely investigated since a large proportion of this radiation is absorbed by the pigments present in green tissues (Lichtenthaler 1996). For instance, bands within the blue (450–520 nm) and green (520–600 nm) channels were found to be sensitive to aboveground biomass in wheat (Wang et al. 2017). In the transition from VS to IR, the so-called red edge, not only the highest correlation between bands and DMY was detected but also a relatively increased density of selected bands. The singularity of this region was also observed in the fact that it was correlated neither with VS nor with IR (Suppl. Fig. S1). Between 680–750 nm, the reflectivity of chlorophyll is sharply increased, a phenomenon that can be used to remotely assess plant health and growth (Seager et al. 2005) as well as chlorophyll concentrations (Filella and Penuelas 1994) and biomass at high canopy densities (Mutanga and Skidmore 2004). Similarly, the IR contains important information about physiological processes affecting biomass including chlorophylls and photosynthesis activity, as well as plant water status (Tucker 1979). The present work included IR data up to ~ 1000 nm, which have been revealed as highly relevant for DMY prediction. Considering that currently there are configurations that allow sensors to collect a broader IR spectrum, further research should focus on the benefits of deploying hyperspectral sensors capable of collecting additional reflectance data up to 2500 nm.

### Improved prediction abilities by combining different sources of information

Under both validation procedures (S1 and S2), models were calibrated in a TRN of increased size. Overall, a positive correlation between the prediction abilities of models and TRN size was observed (Table 2, Fig. 4, Suppl. Fig. S6). The positive influence of TRN size in GS accuracy is well acknowledged in animal (VanRaden et al. 2009) and plant (Marulanda et al. 2015) breeding. Based on our results from the validation scenario S1, this trend also applies to HBLUP, multi-kernel, and bivariate models. Interestingly, the negative impact of reduced TRN was dissimilar across single-kernel models. While in larger TRN, GBLUP was more accurate than HBLUP, the opposite was observed in smaller TRN (Table 2, Suppl. Fig. S6). The reduction in the TRN size to a quarter (from 80 to 20%) represented a decay of about one-half and one-third in the prediction abilities of GBLUP and HBLUP, respectively. The predictive power of HBLUP was substantially higher than linear models fitted with VIs reported in a previous study (Galán et al. 2020). This is in complete agreement with Aguate et al. (2017) and Montesinos-López et al. (2017), who also found the superiority of models based on whole-spectrum data instead of on VIs.

In the validation scenario S2, prediction abilities were lower than in S1, indicating that predicting the yield of genotypes in a new environment is challenging (Fig. 4). In the DAPC (Suppl. Fig. S7), environments within the same year were grouped, reflecting the strong influence of the year effect, not only on agronomic traits (Galán et al. 2020) but also in the hyperspectral data collected at each site. The environmental conditions were very contrasting between 2017 and 2018. In Germany, 2018 was a very dry year, especially on the light sandy soils where rye is usually grown, and our experiments were conducted, e.g., Petkus, which has a very light soil (Suppl. Table S1). In this context, the inclusion of the maximum number of environments in the TRN, leaving only one as a validation environment was beneficial. Under CV accounting for environmental sampling, Utz et al. (2000) also observed that the proportion of the genotypic variance explained by models was enhanced by the inclusion of more environments in the TRN, especially for moderate inherited traits such as GY and GY components. In our study, HBLUP performance was even smaller if the VAL was composed of an environment poorly correlated with the sites within the TRN. Since models in S2 borrow information from closely related environments, prediction of these low correlated environments following this scheme is not recommended.

These findings suggest that the incorporation of hyperspectral data to enhance DMY prediction in rye could improve breeding efficiency. First, if due to budget constraints, a larger TRN size is not affordable, HBLUP could be a valid strategy to precisely predict DMY. Second, the higher prediction ability of multi-kernel and bivariate models indicates that the incorporation of reflectance data and agronomic traits like PH into GS routines has a synergetic effect, when these data are correlated with the target trait. These findings are consistent with Krause et al. (2019), who found that for predicting GY in wheat, single-kernel models fitted with genomic- or hyperspectral-derived relationship matrices yielded similar results but multi-kernel models integrating both matrices surpassed both.

However, our results suggested that the use of bivariate models should be used with caution. On the one hand, they had the highest variability in small TRN. Under these circumstances, the sampling variability is substantially increased. Therefore, the advantage of bivariate over univariate models is reduced. Thus, multivariate regression analysis is not recommended for small sample sizes. On the other hand, the positive correlation between PH and DMY (Fig. 2) suggests that these prediction models should be used with care because taller genotypes would tend to be favored in the selection as indeed observed in Suppl. Table S4. So, breeding for increased lodging resistance would be highly advisable since even small differences in PH of the selected genotypes will multiply when subsequent breeding cycles are contemplated.

It has to be considered that we estimated our prediction abilities within one larger population by fivefold cross-validation. Validation scenario S1 was fitted with environmentally and genetically related data, representing a possible source of bias on the estimation of the predictive power of the models. Further research is needed to predict DMY across genetically different plant materials and/or different selection cycles, i.e., after recombination of selected entries. This would also include results from untested environments that might have a high impact on prediction ability, as shown in Fig. 4.

## Conclusions

While the needs of sustainable renewable energy sources increase, the interest for high-yielding varieties to diversify maize-based cropping systems is boosted in proportion. To meet this demand, novel breeding strategies are needed to fully exploit the potential of rye as a biomass substrate. This study provided strong evidence that hyperspectral data can substantially improve the indirect selection of DMY within the same breeding population, thus enabling a cost-effective dual-purpose program using both DMY and GY as target traits. The reduction in data dimensionality could further enhance the prediction ability of models based on reflectance data. Relationship matrices derived from HTP data could be utilized as an alternative to GS when molecular data are not available, especially under reduced TRN sizes. Additionally, they are a suitable complementary source of information to leverage the accuracy of genomic tools. The superiority of the bivariate model over the multi-kernel model indicates that agronomic traits correlated with DMY can further enhance the efficiency of selection. Similar to the comparison of model performances across different TRN sizes, it would be relevant for practical breeding to investigate prediction ability across a varying degree of relatedness between the TRN and the VAL. Such analysis could assist breeders facing challenging prediction scenarios, including predicting new environments or novel lines that are unrelated to the training population.

## Electronic supplementary material

Below is the link to the electronic supplementary material.

Supplementary file1 (PDF 1113 kb)
