# Supplementary material for: Integration of genotypic, hyperspectral, and phenotypic data to improve biomass yield prediction in hybrid rye
Source: Theor Appl Genet. 2020 Jul 17;133(11):3001–15. doi: 10.1007/s00122-020-03651-8 (PMC7548001; doi:10.1007/s00122-020-03651-8)
Supplement: Supplementary file 1 — Supplementary file1 (PDF 1113 kb) [file 122_2020_3651_MOESM1_ESM.pdf]

Journal:

Theoretical and Applied Genetics

Title:

Integration of genotypic, hyperspectral, and phenotypic data to improve biomass yield prediction in hybrid rye

Authors:

Rodrigo José Galán[1], Angela-Maria Bernal-Vasquez[2], Christian Jebsen[2], Hans-Peter Piepho[3], Patrick Thorwarth[1,2], Philipp Steffan[4], Andres Gordillo[4], Thomas Miedaner[1].

Affiliation:

[1] State Plant Breeding Institute, University of Hohenheim, 70593 Stuttgart, Germany.

[2] KWS SAAT SE, Grimsehlstraße 31, 37574 Einbeck, Germany.

[3] Biostatistics Unit, Institute of Crop Science, University of Hohenheim, 70593 Stuttgart, Germany.

[4] KWS LOCHOW GMBH, Ferdinand-von-Lochow Straße 5, 29303 Bergen, Germany.

Corresponding author:

Thomas Miedaner
e-mail: thomas.miedaner@uni-hohenheim.de
Telephone No.: +49 711 459-22690

Available ORCID of the authors:

Angela-Maria Bernal-Vasquez: https://orcid.org/0000-0003-0415-831

Patrick Thorwarth: https://orcid.org/0000-0003-4456-2358

Thomas Miedaner: https://orcid.org/0000-0002-9541-3726

Online resource 1

Supplementary Table S1 Characterization of the four locations in Germany.

Location	Coordinates	Altitude (m a.s.l.)	Soil type	pH	P_2O_5 [mg/100g soil] [a]	K_2O [mg/100g soil] [a]	Mg [mg/100g soil] [a]	OM [%][a]	Nmin [kg/ha] [a]	Growing-Season Precipitation (mm) [b]	Growing-Season Average temperature (°C) [b]	Sowing dates	Harvest dates	Flight dates
Bernburg	51°49'37.66N 11°43'37.15"E	85	Black soil					--------------------- 2017 ---------------------						
				7.4	26	26	8	2	60	203	8.6	Sept. 30 2016	June 15 2017	May 23 2017
								--------------------- 2018 ---------------------						
				7.4	25	33	8	2	44	300	7.2	Sept. 29 2017	June 6 2018	May 28 & June 06 2018
Petkus	51°59'2.61"N 13°21'29.38"E	137	Sandy loam					--------------------- 2017 ---------------------						
				6.48	13.2	5.22	7.21	-	36	319	6.2	Oct. 10 2016	June 20 2017	June 08 & June 18 2017
								--------------------- 2018 ---------------------						
				6.48	1.2	5.71	4.43	1.51	33	258	8.2	Oct 17 2017	June 8 2018	May 31 & June 07 2018
Wohlde	52°48'36.01"N 10°0'46.73"E	77	Loamy sand					--------------------- 2017 ---------------------						
				5.7	7	10.8	8	-	45	440	7.1	Sept. 29 2016	June 21 2017	May 19 & June 19 2017
								--------------------- 2018 ---------------------						
				5.8	7	14.8	4.7	-	25	410	8.05	Oct. 17 2017	June 13 2018	May 25 and June 05 2018
Prislich	53°15'58.73"N 11°39'36.11"E	38	sandy loam					--------------------- 2017 ---------------------						
				5.8	4.2	16	6	-	32	180	13.8	Oct. 10 2016	June 21 2017	June 09 and June 20 2017
								--------------------- 2018 ---------------------						
				5.9	4.5	19	3	-	4	137	7.8	Sept. 20 2017	June 14 2018	May 29 & June 12 2018

[a] Soil condition: Phosphorus Pentoxide P_2O_5 [mg/100g soil]; Potassium oxide K_2O [mg/100g soil]; Magnesium Mg [mg/100g soil]; Organic matter OM [%]; Nitrogen concentration in the 0-60 cm soil layer Nmin [kg/ha].

[b] Weather data is incomplete due to technical problems in the weather stations in Bernburg (Oct.-Dec. 2016) and Prislich (Oct 2016-March 2017).

Supplementary Table S2 Hyperspectral bands (WL) selected (recovery rate > 40%) by the least absolute shrinkage and selection operator (Lasso) for 274 winter rye hybrids assessed in eight environments and two flight dates.

Visible Spectrum [a]	Infrared radiation [a]
WL410, WL414, WL565, WL588, WL673, WL680	WL702, WL721, WL723, WL733, WL739, WL742, WL758, WL759, WL761, WL764, WL825, WL854, WL908, WL917, WL932, WL935, WL939, WL942, WL945, WL959, WL968, WL970, WL978, WL980, WL987, WL993

[a] WLλ is the reflectance at a specific wavelength λ (±1 nm at the center of the band)

Supplementary Table S3 Mean prediction abilities and standard errors for dry matter yield of six models across different training set sizes determined for 274 winter rye hybrid assessed in eight environments. Hyperspectral data was collected on two flight dates (“first” and “second”), which were separately analyzed.

Model [a]	Flight date	Training set size [b]			
		20%	40%	60%	80%
HBLUP	First	0.41^{g} ± 0.004	0.49^{e} ± 0.002	0.53^{f} ± 0.002	0.54^{de} ± 0.004
	Second	0.44^{ef} ± 0.003	0.49^{e} ± 0.002	0.51^{g} ± 0.003	0.52^{ef} ± 0.004
G+H	First	0.42^{fg} ± 0.003	0.56^{d} ± 0.002	0.64^{d} ± 0.002	0.68^{c} ± 0.003
	Second	0.48^{d} ± 0.003	0.59^{c} ± 0.002	0.65^{c} ± 0.002	0.70^{b} ± 0.003
Bivariate_G	First	0.53^{bc} ± 0.004	0.61^{b} ± 0.002	0.67^{bc} ± 0.003	0.68^{c} ± 0.003
	Second	0.54^{ab} ± 0.003	0.65^{a} ± 0.003	0.67^{bc} ± 0.003	0.68^{c} ± 0.003
Bivariate_H	First	0.45^{e} ± 0.005	0.50^{e} ± 0.004	0.55^{e} ± 0.003	0.55^{d} ± 0.004
	Second	0.44^{ef} ± 0.006	0.48^{e} ± 0.006	0.49^{g} ± 0.005	0.50^{f} ± 0.005
Bivariate_G+H	First	0.52^{c} ± 0.007	0.61^{b} ± 0.005	0.68^{b} ± 0.003	0.72^{b} ± 0.003
	Second	0.56^{a} ± 0.007	0.65^{a} ± 0.004	0.70^{a} ± 0.004	0.75^{a} ± 0.002

[a] See Table 1 for more information about the listed models.

[b] Within a column, mean values followed by no letter in common are significantly different (Tukey’s honestly significant difference test; α =0.01%).

Supplementary Table S4 Prediction ability (r), dry matter yield (DMY, dt ha^{-1}), and plant height (PH, cm) obtained at different selected fractions by prediction models (TRN=80%) fitted with 274 winter rye hybrids assessed in eight environments and two flight dates.

Model [a]	Criterium	Selected fraction								$\bar{x}_{overall}$ [b]
		10%		20%		30%		40%		
		$\bar{x}$	SE	$\bar{x}$	SE	$\bar{x}$	SE	$\bar{x}$	SE	
GBLUP	r	0.33	0.02	0.35	0.01	0.43	0.01	0.46	0.01	0.39 b
HBLUP	r	0.25	0.02	0.31	0.01	0.38	0.01	0.42	0.01	0.34 c
G+H	r	0.30	0.02	0.39	0.01	0.43	0.01	0.47	0.01	0.40 b
Bivariate_G	r	0.42	0.01	0.44	0.01	0.49	0.01	0.51	0.01	0.47 a
Bivariate_H	r	0.25	0.02	0.36	0.01	0.41	0.01	0.40	0.01	0.36 c
Bivariate_G+H	r	0.33	0.02	0.45	0.01	0.48	0.01	0.50	0.01	0.44 a
GBLUP	DMY	122.30	0.04	121.76	0.03	121.21	0.02	120.88	0.02	121.54 (+1,7%) d
HBLUP	DMY	122.23	0.04	121.77	0.03	121.31	0.02	120.98	0.02	121.57 (+1,8%) d
G+H	DMY	122.64	0.04	122.04	0.03	121.59	0.02	121.26	0.02	121.89 (+2,0%) b
Bivariate_G	DMY	122.67	0.04	121.99	0.03	121.41	0.02	121.07	0.02	121.79 (+1,9%) c
Bivariate_H	DMY	122.31	0.04	121.73	0.03	121.30	0.02	121.05	0.02	121.60 (+1,8%) d
Bivariate_G+H	DMY	122.84	0.03	122.13	0.03	121.65	0.02	121.33	0.02	121.99 (+2,1%) a
GBLUP	PH	119.24	0.06	118.57	0.04	117.95	0.04	117.64	0.03	118.35 (+1,7%) e
HBLUP	PH	119.10	0.05	118.74	0.04	118.36	0.03	118.06	0.03	118.57 (+1,9%) d
G+H	PH	119.63	0.05	119.00	0.04	118.56	0.03	118.26	0.03	118.86 (+2,2%) c
Bivariate_G	PH	120.34	0.06	119.33	0.05	118.72	0.03	118.36	0.03	119.19 (+2,5%) b
Bivariate_H	PH	119.92	0.06	119.37	0.04	118.83	0.03	118.41	0.03	119.13 (+2,4%) b
Bivariate_G+H	PH	120.42	0.06	119.63	0.04	119.08	0.03	118.74	0.03	119.47 (+2,7%) a

[a] See Table 1 for more information about the listed models.

$\bar{x}$, mean; SE, standard error of the mean; $\bar{x}_{overall}$, mean across all selected fractions.

[b] The percent difference between the mean across all selected fractions and the population mean (DMY=119.48 dt ha^{-1}; PH=116.34 cm) is listed in brackets. Mean values followed by no letter in common are significantly different (Tukey's honestly significant difference test; α =0.01%).

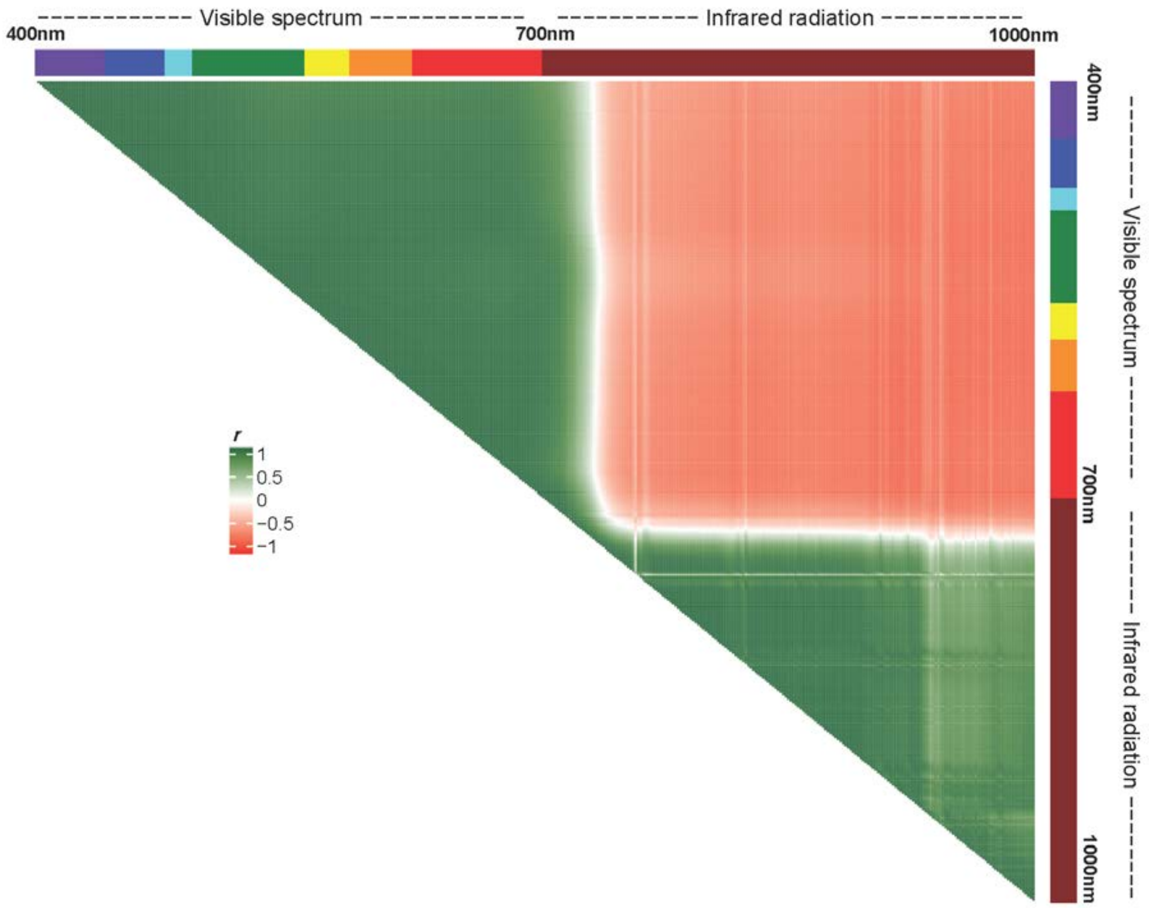

Supplementary Fig. S1 Pearson's coefficients of correlation (r) across hyperspectral bands based on 274 rye hybrids across eight environments.

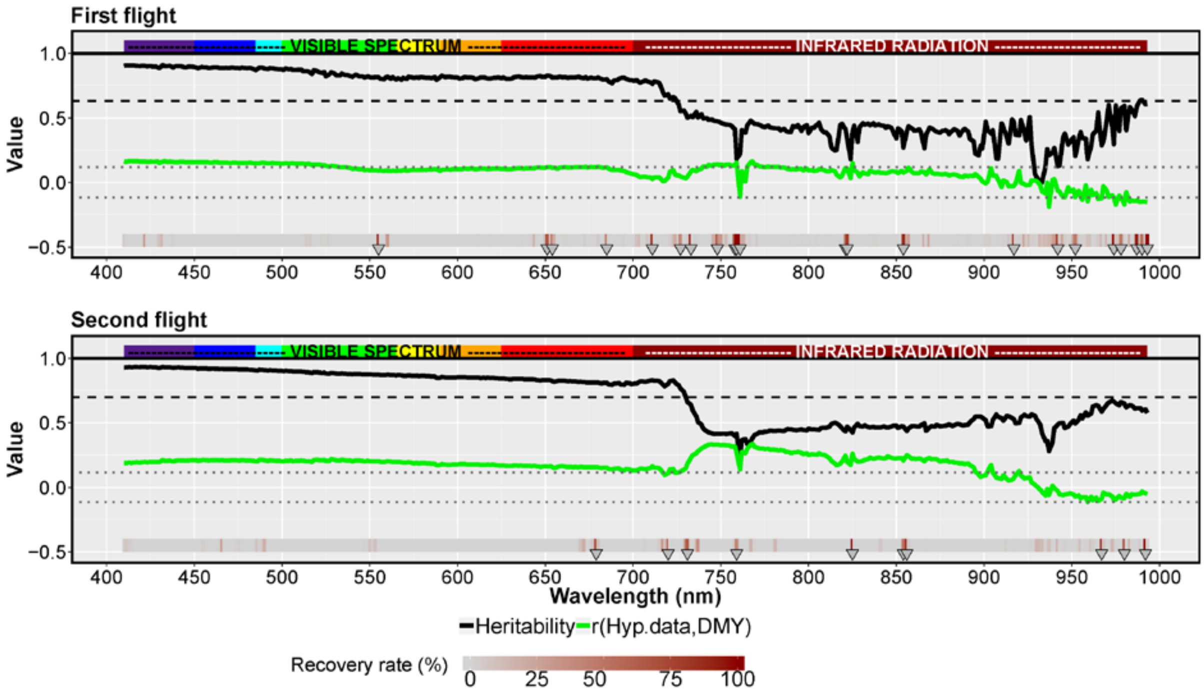

Supplementary Fig. S2 Heritability estimates (black line) for the hyperspectral bands, phenotypic correlations (r, green line) between hyperspectral bands and dry matter yield, and recovery rate (%) of hyperspectral bands after the least absolute shrinkage and selection operator (Lasso, gray-red heatmap) for 274 winter rye hybrids assessed in eight environments shown by flight date. The mean heritability across all wavelengths is denoted by the dashed black line. Correlation values $\geq |0.12|$ are significant ($p<0.05$) as showed by the gray dotted lines. Selected hyperspectral bands (recovery rate > 40%) are indicated by the gray triangles.

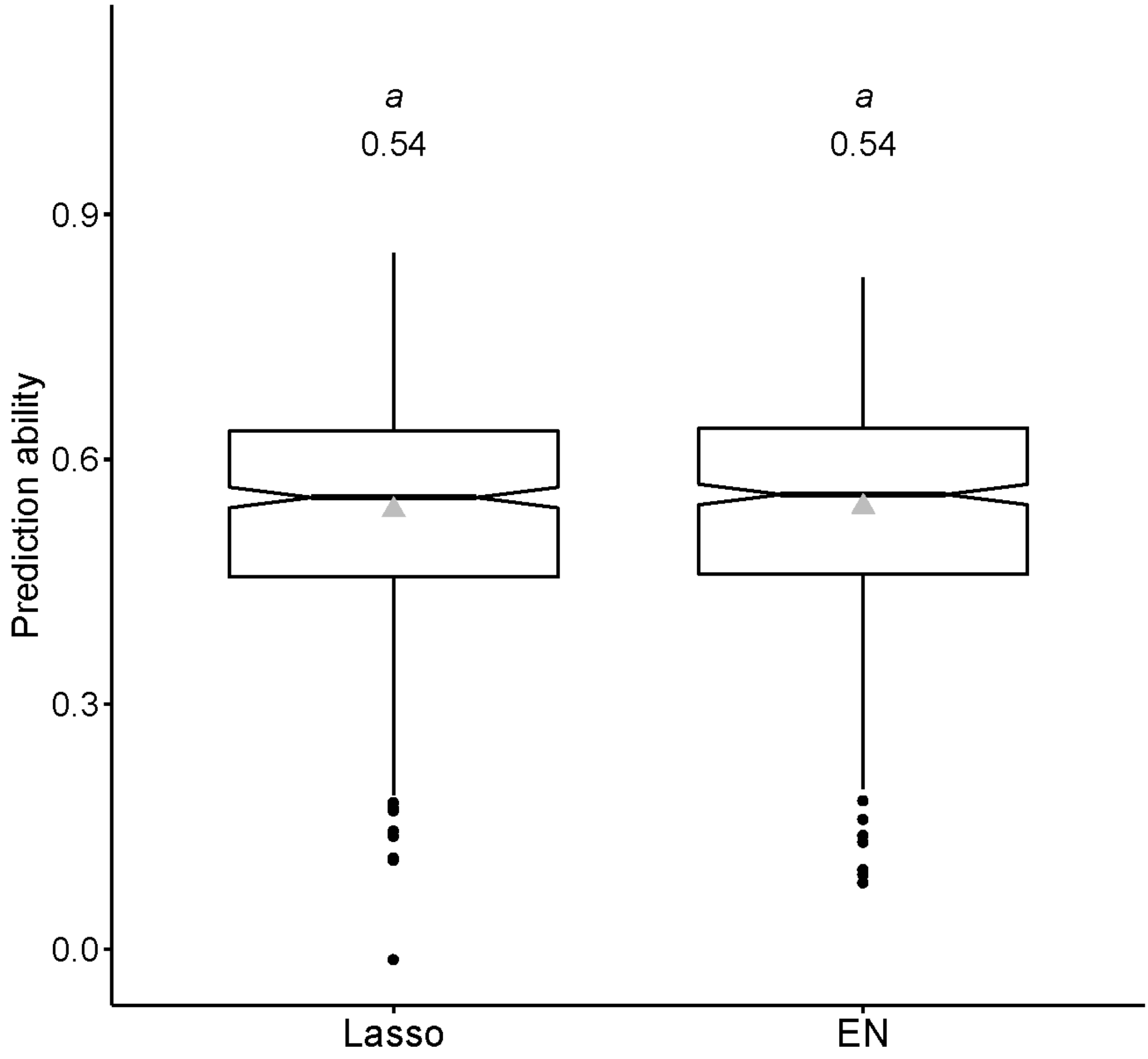

Supplementary Fig. S3 Prediction ability for dry matter yield based the least absolute shrinkage and selection operator (Lasso) and elastic net (EN) fitted with hyperspectral data collected in eight environments and two flight dates for 274 winter rye hybrids. Mean values are shown above each box plot and by gray triangles and are significantly different when headed by no letter in common (Tukey's honestly significant difference test; α =0.01%).

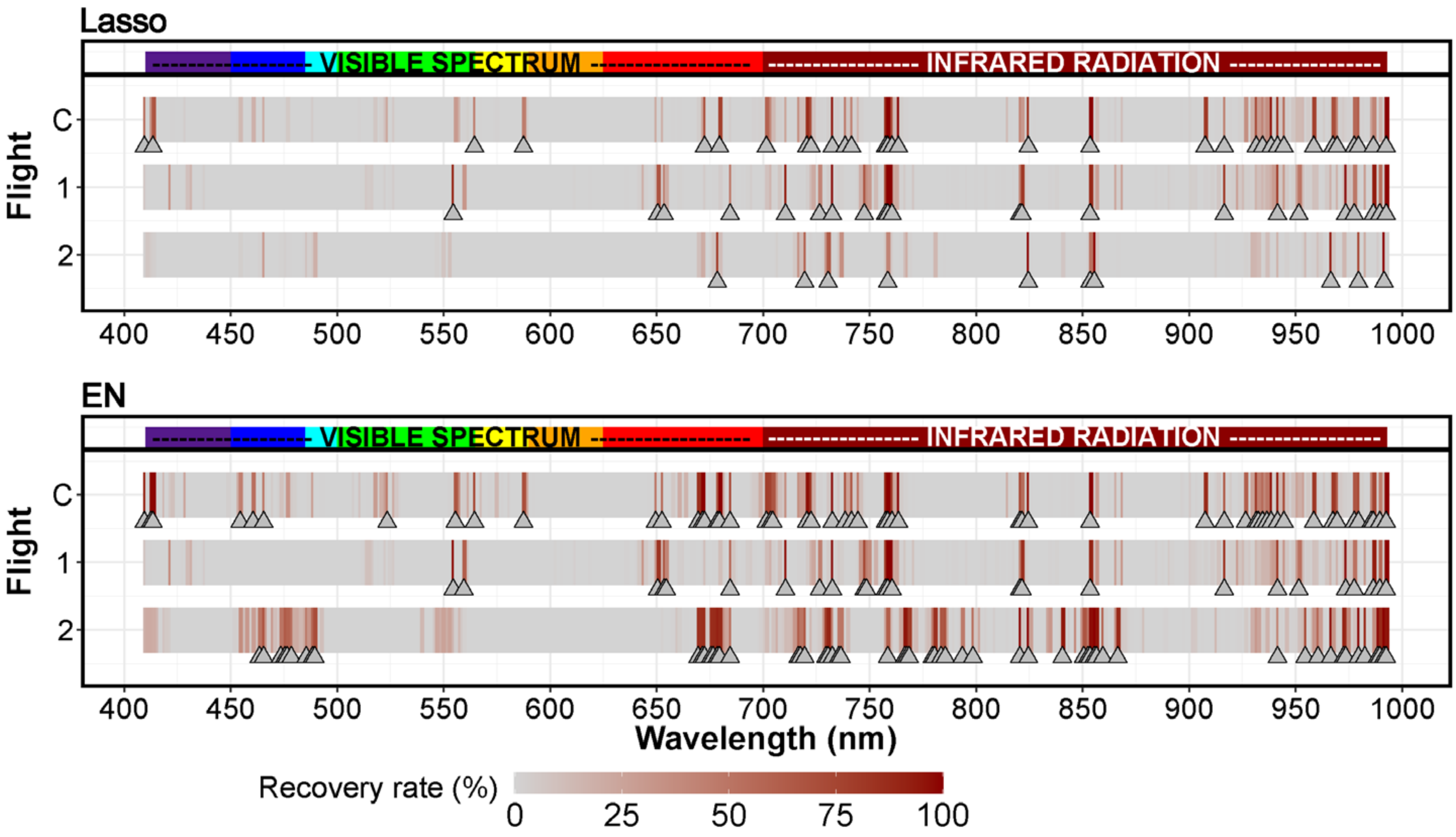

Supplementary Fig. S4 Hyperspectral bands selected (recovery rate > 40%) by the least absolute shrinkage and selection operator (Lasso) and elastic net (EN) fitted with hyperspectral data collected for 274 winter rye hybrids assessed in eight environments and two flight dates (1; First, 2; Second), which were individually and combined (C) analyzed.

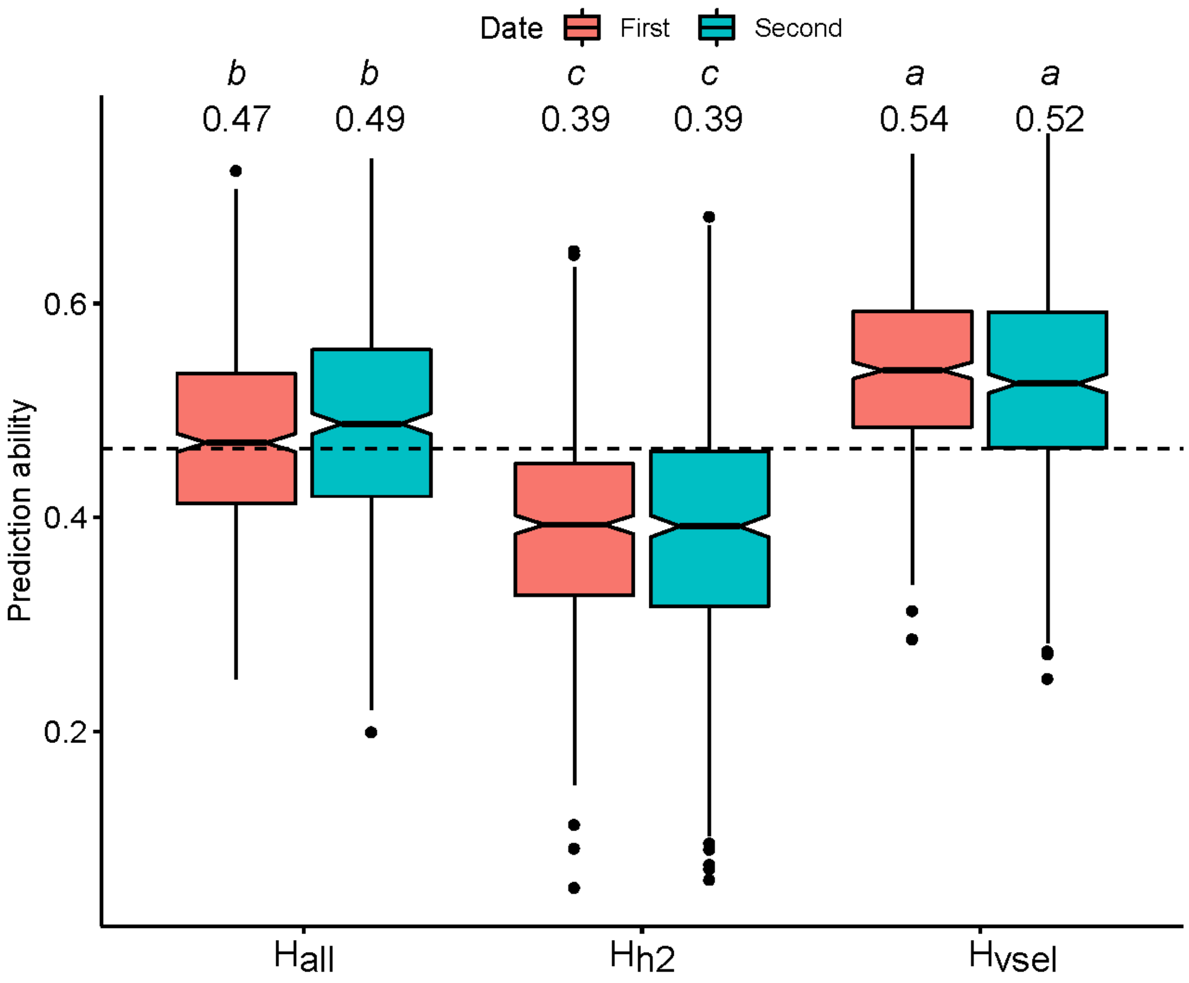

Supplementary Fig. S5 Prediction ability for dry matter yield of hyperspectral best linear unbiased predictor model (HBLUP) collected on single flight dates based on different **H** relationship matrices, including all available 400 bands ($\mathbf{H}_{all}$), bands with heritability > mean heritability across wavelengths **($\mathbf{H}_{h2}$)**, and only selected bands ($\mathbf{H}_{vsel}$) for 274 winter rye hybrids. Mean values are shown above each box plot and are significantly different when headed by no letter in common (Tukey's honestly significant difference test; α =0.01%). The mean prediction ability across all models and flight dates is denoted by the dashed black line.

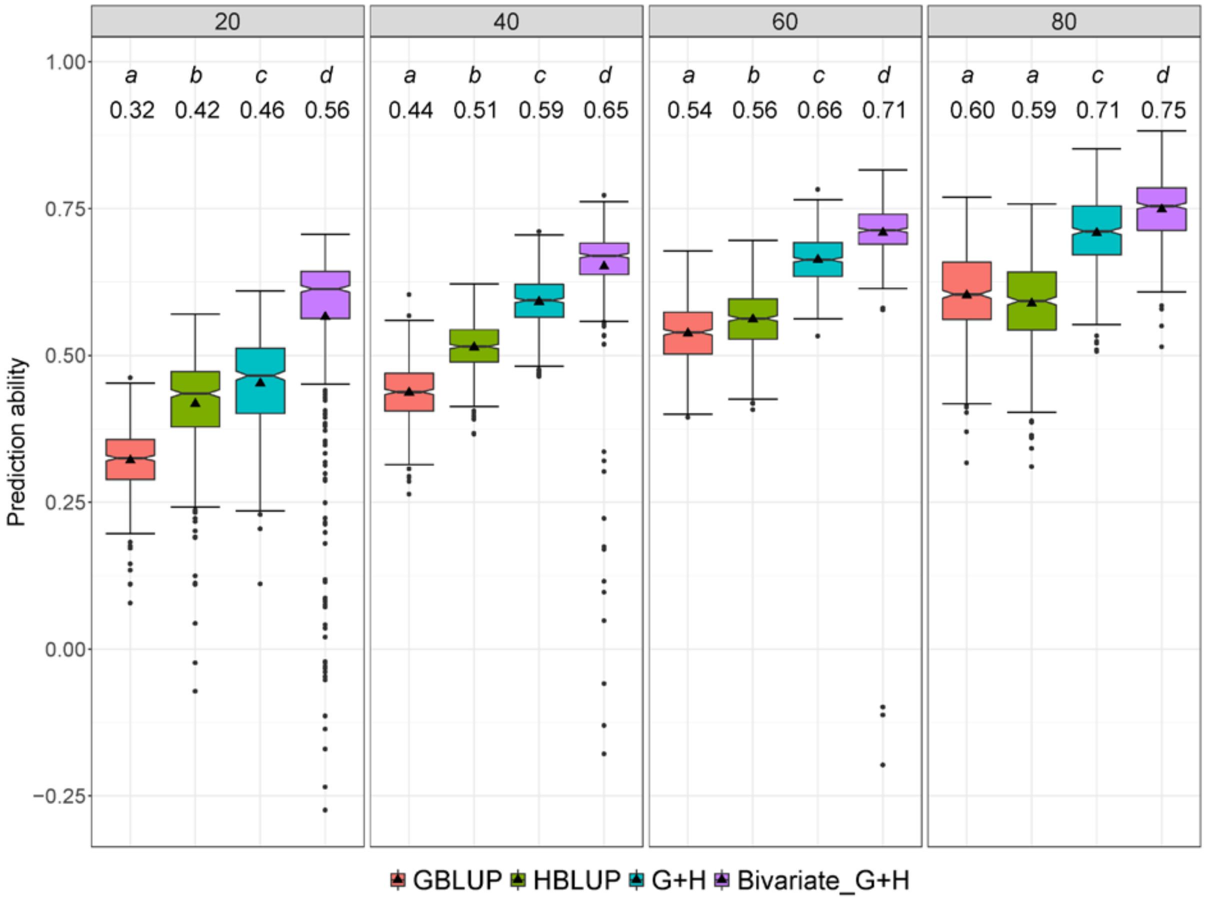

Supplementary Fig. S6 Prediction ability for dry matter yield of single-kernel (Genomic best linear unbiased predictor, GBLUP and Hyperspectral best linear unbiased predictor, HBLUP), multi-kernel (G+H), and bivariate (Bivariate_G+H) models trained in four different training set (TRN) sizes for 274 winter rye hybrids. Models were tested under **validation scenario S1.** TRN sizes in percentage are shown at the top of each subplot. Mean values are shown above each box plot and by black triangles. Means headed within the same TRN size by no letter in common are significantly different (Tukey's honestly significant difference test; α =0.01%).

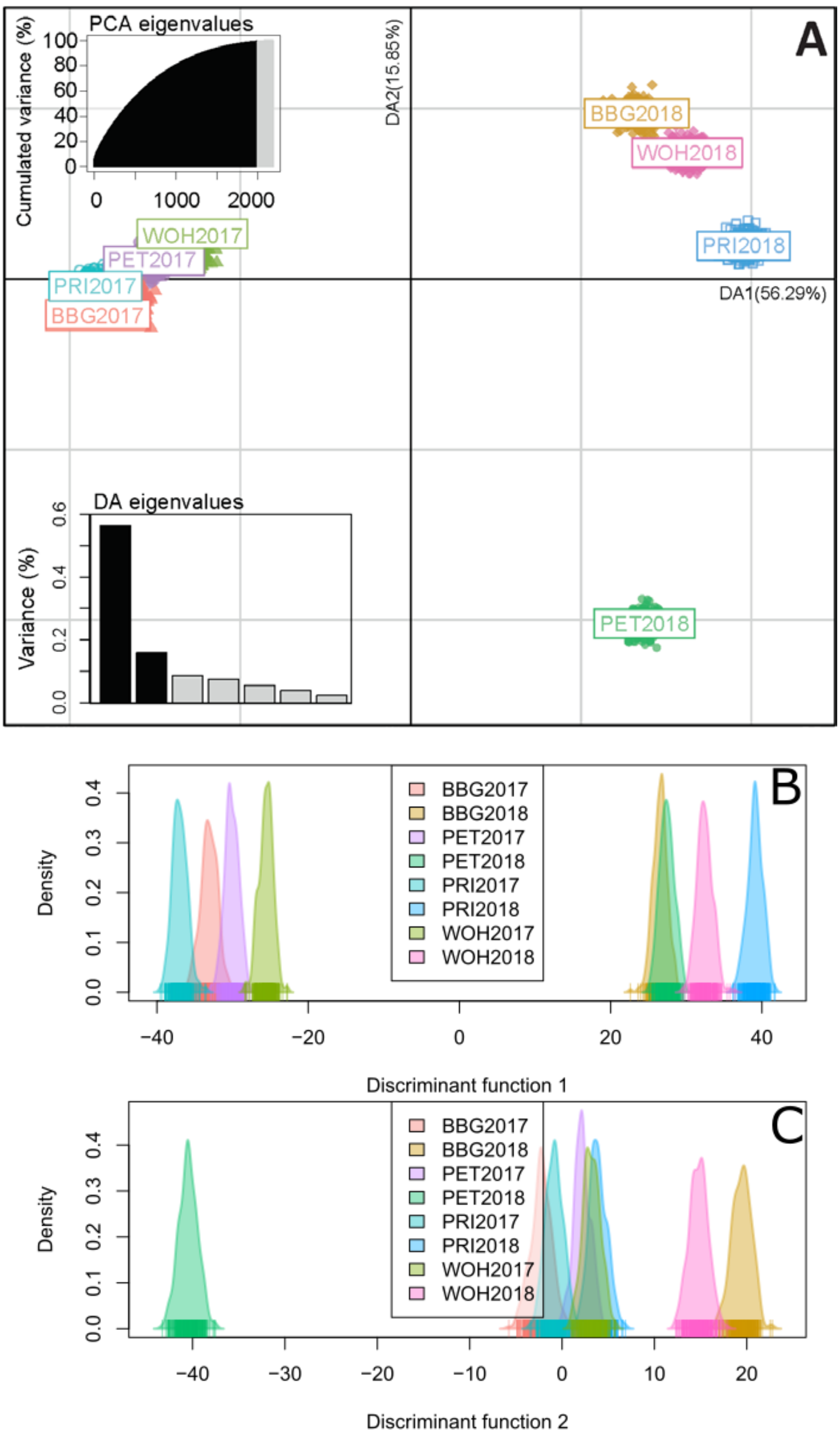

Supplementary Fig. S7 Discriminant analysis of principal components (DAPC) showing the clustering pattern of 274 winter rye hybrids across eight environments based on hyperspectral reflectance data. Scatter plot (A) for the first two discriminant functions (DA) including at the top left the PCA eigenvalues retained (in black) and at the bottom left the variation explained by each DA eigenvalues. Densities of individuals on the first (B) and the second (C) DA are also displayed.
